# Recent Advances in Photoacoustic Tomography

**DOI:** 10.34133/2021/9823268

**Published:** 2021-05-28

**Authors:** Lei Li, Lihong V. Wang

**Affiliations:** Caltech Optical Imaging Laboratory, Andrew and Peggy Cherng Department of Medical Engineering, Department of Electrical Engineering, California Institute of Technology, 1200 East California Boulevard, Mail Code 138-78, Pasadena, CA 91125, USA

## Abstract

Photoacoustic tomography (PAT) that integrates the molecular contrast of optical imaging with the high spatial resolution of ultrasound imaging in deep tissue has widespread applications in basic biological science, preclinical research, and clinical trials. Recently, tremendous progress has been made in PAT regarding technical innovations, preclinical applications, and clinical translations. Here, we selectively review the recent progresses and advances in PAT, including the development of advanced PAT systems for small-animal and human imaging, newly engineered optical probes for molecular imaging, broad-spectrum PAT for label-free imaging of biological tissues, high-throughput snapshot photoacoustic topography, and integration of machine learning for image reconstruction and processing. We envision that PAT will have further technical developments and more impactful applications in biomedicine.

## 1. Introduction

Biomedical imaging has played a significant role in modern medicine for diagnosing diseases, monitoring therapy, and providing biological insights into lives [1, 2]. Photoacoustic tomography (PAT), also known as optoacoustic tomography, is an emerging biomedical imaging modality that provides cross-sectional or three-dimensional (3D) imaging of an object based on the photoacoustic (PA) effect—a physical phenomenon that converts absorbed light to sound [3]. Although discovery of the photoacoustic effect dates back to 1880 [4], PAT has enjoyed a rapid growth since early 2000s following the development in ultrasonic detectors, computations, and lasers [5]. Since 2010, PA imaging has become one of the largest research areas in biophotonics and still enjoys the rapid growth [6].

**Figure 1:**
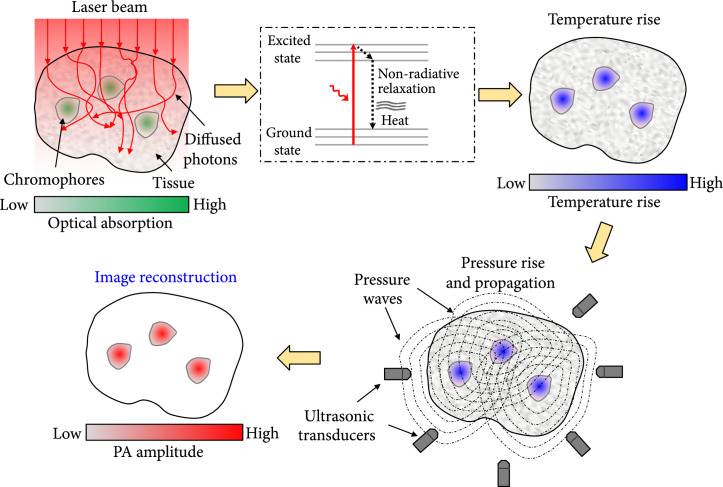
The principle of PAT.

Figure 1 illustrates the principle of PAT. Typically, PAT employs nonionizing laser pulses directed to the object for excitation. When using microwave or radiofrequency pulses for illumination, it is referred to as thermoacoustic tomography (TAT) [7, 8]. The delivered photons are absorbed by the molecules in the object, elevating the molecules from the ground state to the excited state. Another photon or heat is emitted, when the excited molecule relaxes to the ground state. The absorbed photons can be converted to heat through nonradiative relaxation [9]. Via thermoelastic expansion, a pressure rise is induced by the heat [10]. The pressure rise propagates (with a speed of ~1500 m/s) inside biological tissue as an acoustic wave, also mentioned as a PA wave [11, 12]. By detecting the PA waves, we can form images that map the optical absorption of the object. Based on the image formation methods, PAT has two major implementations: PA microscopy (PAM) and PA computed tomography (PACT) [6]. PAM forms images by raster scanning the focus of light and sound across the object, while PACT yields images by inverse reconstruct the detected signals induced by wide-field illumination.

**Figure 2:**
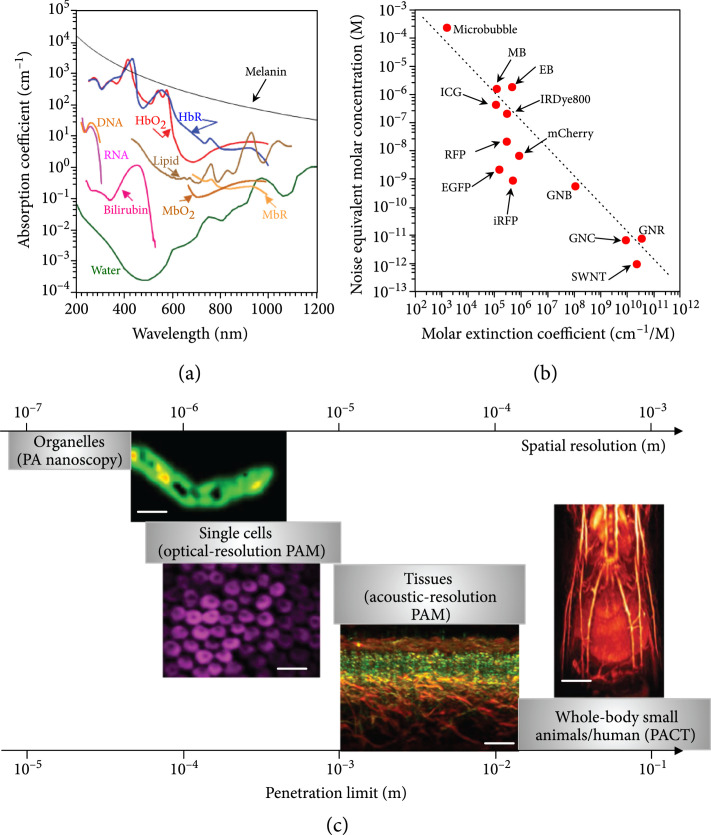
Multicontrast and multiscale PAT. (a) Absorption spectra of endogenous molecules at normal concentrations *in vivo* [27]. Bilirubin: 12 mg L^-1^ in blood; DNA/RNA: 1 g L^-1^ in cell nuclei; HbO_2_: oxyhemoglobin; HbR: deoxyhemoglobin, 2.3 mM in blood; MbO_2_: oxymyoglobin; MbR: reduced myoglobin, 0.5% mass concentration in skeletal muscle; melanin: 14.3 g L^-1^ in the skin; lipid: 20% volume concentration in tissue; water: 80% volume concentration in tissue. (b) Noise equivalent molar concentrations of some widely used exogenous contrast agents, based on reported values from the literature [27]. Illumination fluence is not compensated. EB: evens blue [45]; EGFP: enhanced green fluorescent protein [46]; GNB: gold nanobeacon [47]; GNC: gold nanocage [48]; GNR: gold nanorod [49]; ICG: indocyanine green [50]; IRDye800: near-infrared Dye800 [51]; iRFP: near-infrared red fluorescent protein [52]; MB: methylene blue [53]; mCherry: monomeric cherry protein [46]; microbubble [54]; RFP: red fluorescent protein [52]; SWNT: single-walled nanotube [55]. The dashed curve is power function fitting $y=0.1x^{-1}$, where y is the noise equivalent concentration in molars and x the molar extinction coefficient in cm^-1^ M^-1^. (c) Multiscale PAT and representative images. Organelles and PA nanoscopy of a single mitochondrion (scale bar, 500 nm) [37]. Single cells, optical-resolution PAM of red blood cells (scale bar, 20 *μ*m) [38]. Tissues, acoustic-resolution PAM of human skin (scale bar, 500 *μ*m) [25]. Whole-body small animals and whole-body PACT of a nude mouse *in vivo* (scale bar, 4 mm) [39].

PAT is a hybrid imaging method that harvests both optical and acoustic energies. Thus, PAT inherits the advantages of both optical and ultrasound imaging, offering rich optical contrast and high spatial resolution inside deep tissue [13]. PAT is directly sensitive to molecules’ optical absorption [14]. By exciting molecules at preferred wavelengths according to the spectrum signatures, PAT achieves multicontrast imaging of molecules based on their chemical compositions [15]. Till now, PAT has spectroscopically imaged numerous endogenous molecules, including oxy- and deoxy-hemoglobin, oxy- and reduced myoglobin, melanin, cytochrome, DNA/RNA, bilirubin, lipid, and water, which enables PAT for anatomical, functional, metabolic, and histologic imaging (Figure 2(a)) [16-27]. Thanks to the strong optical absorption of exogenous contrast agents, e.g., micro/nanoparticles, organic dyes, and genetically encoded proteins, PAT enjoys superb sensitivity in deep tissue and offers molecular imaging (Figure 2(b)) [27-33]. Increasing the optical fluence improves the detection sensitivity, as long as the temperature increase is within the safety limit. To guarantee the safety, typically, the laser exposure to the skin surface and eye is regulated by the American National Standards Institute (ANSI) standard. Thanks to the PA effect, PAT detects ultrasonic waves induced by excitation photons, including both ballistic and scattered ones; thus, PAT achieves much deeper penetration than conventional optical microscopy relying on ballistic photons. Moreover, acoustic waves are orders of magnitude less scattered inside soft tissues; therefore, PAT provides far better spatial resolution than pure optical imaging methods in deep tissues (>2 mm) [34]. With a skin surface radiant exposure of 21 mJ/cm^2^ at 1064 nm, which is only one-fifth of the ANSI limit (100 mJ/cm^2^), PAT demonstrated an imaging thickness up to 7 cm *in vivo* with double-sided light illumination [35]. Because of the acoustic detection, PAT’s spatial resolution and penetration depth are scalable with the detected acoustic frequency. The spatial resolution of PAT improves as the acoustic central frequency and bandwidth increase at the expense of penetration depth [36]. PAT has uniquely shown a capability of multiscale imaging using a consistent optical contrast, ranging from organelles, cells, and tissues to whole-body small animals and human organs, as shown in Figure 2(c) [25, 37-39]. Previously, preclinical small-animal imaging and clinical applications typically employ nonoptical imaging modalities, including magnetic resonance imaging (MRI), X-ray computed tomography (X-ray CT), positron emission tomography (PET), single-photon emission computed tomography (SPECT), and ultrasound imaging (USI), which all can provide deep penetration [40]. However, those approaches still face challenges. For example, MRI necessitates a long data-acquisition time, not suitable for capturing fast dynamics [41, 42]. PET and SPECT have a low spatial resolution to resolve detailed structures. X-ray CT, PET, and SPECT employ ionizing radiation, impeding longitudinal monitoring [43]. Conventional USI does not reveal molecular contrasts outside blood vessels [44]. PAT, as a noninvasive approach, achieves high-resolution imaging in deep tissues with optical contrasts, providing a complementary approach for preclinical research and clinical translations.

In recent years, PAT has an even more rapid development, including technical innovations, various biomedical applications, and clinical translations. In this paper, we selectively review some of the recent progresses and advances in PAT, including small-animal whole-body imaging, novel molecular imaging, rapid assessments of brain functions, broad-spectrum imaging of tissues, human organ imaging, snapshot photoacoustic topography, and integration of machine learning for advanced image reconstruction. We envision that PAT will have more impactful applications in fundamental science, preclinical research, and clinical trials.

## 2. Whole-body PACT of Small Animals

**Figure 3:**
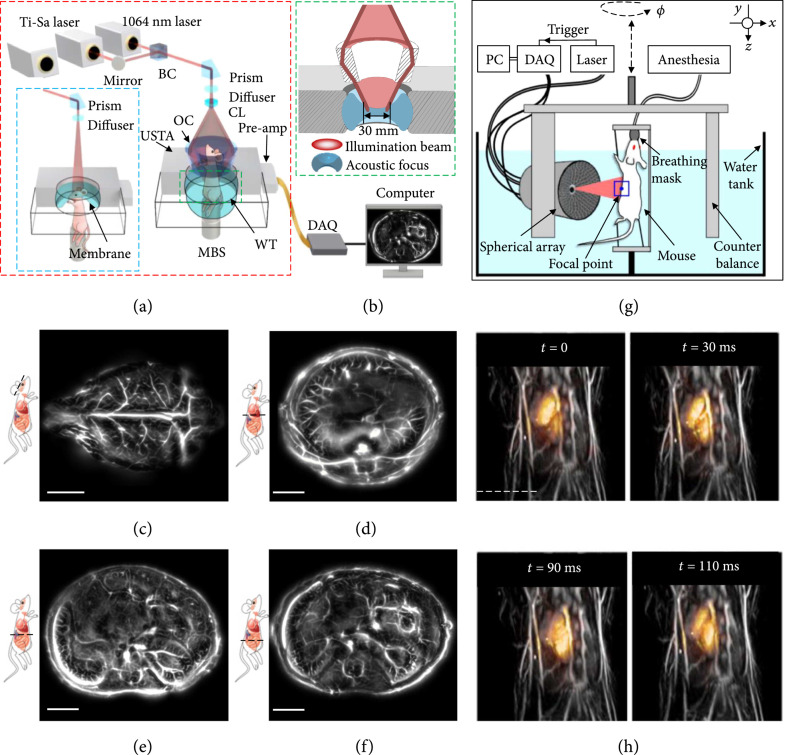
Whole-body PACT of small animals [58, 59]. (a) Schematic of the SIP-PACT system for trunk and brain (blue dashed boxed inset) imaging [58]. Dual-wavelength illumination is used. BC: beam combiner; CL: conical lens; DAQ: data acquisition system; MBS: magnetic base scanner; OC: optical condenser; USTA: (full-ring) ultrasonic transducer array; WT: water tank. (b) Close-up of the green dashed line in (a), showing the confocal design of light illumination and acoustic detection. (c-f) Representative cross-sectional images of the brain (c), the liver (d), the upper abdominal cavity (e), and the lower abdominal cavity (f) in a live mouse, acquired by SIP-PACT [58]. Scale bar: 5 mm. (g) Layout of the spiral scanning PACT system for small-animal whole-body imaging [59]. DAQ: data acquisition unit. (h) Representative 3D whole-body images of a live mouse. Each image overlays the beating mouse heart (color) onto a whole-body anatomical image (gray) of the same mouse [59]. Scale bar: 5 mm.

Small-animal whole-body imaging plays an indispensable role in preclinical study [56]. With optical contrasts and high spatiotemporal resolution, small-animal imaging can provide physiological understandings of biological processes and dynamics, advancing fundamental biology, preclinical study, and clinical translation [57]. A recent development in PACT for small-animal whole-body imaging, termed single-impulse panoramic PACT (SIP-PACT), permits anatomical, functional, and molecular whole-body imaging with exceptional quality and speed [58]. The schematic of SIP-PACT is shown in Figure 3(a). SIP-PACT employed full-ring ultrasonic detection (512 elements, 5 MHz central frequency, over 90% one-way bandwidth) with parallel amplification and digitization, maximizing the detection signal-to-noise ratio (SNR) and speed. The light illumination and acoustic detection are aligned confocally to optimize the detection sensitivity (Figure 3(b)). It takes 50 *μ*s for SIP-PACT to acquire a 2-dimensional (2D) image of a cross-section *in vivo*. The in-plane panoramic acoustic detection of SIP-PACT offered an isotropic resolution (~125 *μ*m) within the whole cross-section and full-view fidelity. Moreover, to better reveal detailed structures of internal organs, a half-time dual-speed-of-sound universal back-projection algorithm was developed to account for the acoustic inhomogeneity between the animal tissue and the surrounding coupling medium (water). Representative small-animal whole-body images, acquired at 1064 nm, from SIP-PACT are shown in Figures 3(c)-3(f) [58].

Recently, a hemispherical transducer array-based PACT system (Figure 3(g)) has been built to image the whole body of small animals [59]. By spiral scanning the hemispherical transducer array (256 elements, 4 MHz central frequency, 100% one-way bandwidth) around the animal, volumetric whole-body imaging can be achieved (Figure 3(g), the whole-body tissue is shown in gray; illumination wavelength, 800 nm). By parking the array at a given position and acquiring data continuously, high-speed imaging of the dynamics inside an organ can be realized. For example, the high imaging speed reveals cardiac dynamics *in vivo* in detail, as shown in Figure 3(h) (the beating heart is shown in color) [59].

The recent progress in small-animal PACT promises more extensive preclinical studies, particularly when high spatiotemporal resolution and high sensitivity are necessary. For instance, tracking immune cells can visualize immune responses during cancer progression and treatment, advancing our understanding of immune system dynamics. Overall, the development of small-animal PACT enables real-time imaging of biological processes in basic life science research and ultimately in clinical applications.

## 3. Molecular PACT

**Figure 4:**
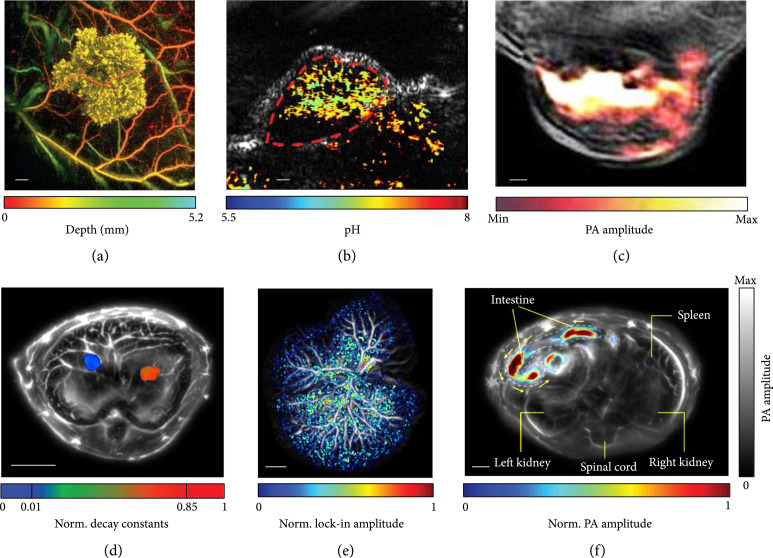
Representative images of molecular PACT. (a) *In vivo* PA images of Tyr-expressing K562 cells after subcutaneous injection into the flank of a nude mouse (vasculature is color-coded for depth; K562 cells are false-colored yellow). Scale bar, 1 mm [64]. (b) Quantitative PACT of pH *in vivo*. Functional PA image in pseudo-color is superimposed on the gray-scale ultrasound image. Scale bar, 2 mm [72]. (c) *In vivo* PA image of a 4T1 tumor-bearing mouse, given a single injection of 150 *μ*g of OMV^Mel^ via the tail vein. The image was acquired at 3 h postinjection, showing the accumulation of OMV^Mel^ in tumor tissue, where the OMV^Mel^ is in color and the background tissue is in gray [73]. (d) *In vivo* multicontrast PACT of two types of tumor cells in the liver. Two types of tumors expressing different photoswitchable proteins are separated by their decay characteristics. The tumors are shown in color, and the background tissues are shown in gray. Norm.: normalized [74]. (e) PA image of a hydrodynamic-transfected liver. The photoswitching signals are shown in color, confirming the existence of reconstituted DrSplit induced by protein-protein interactions. The background tissues are shown in gray [74]. (f) PA image of the microrobots in the intestines *in vivo*. The migrating microrobots are shown in color, and the mouse tissues are shown in gray. The yellow arrows indicate the direction of migration [76].

Because endogenous contrast agents often lack specificity or sensitivity, scientists usually rely on exogenous contrast agents that can visualize biological phenomena with high sensitivity and specificity to study biological activities. Exogenous contrast agents for deep-penetrating PAT hold two key advantages: (1) they are optimized for high optical absorption at near-infrared (NIR) wavelengths for high detection sensitivity inside deep tissues; (2) they are specifically designed to conjugate with targeting moieties to selectively bind to receptors to achieve high specificity. Exogenous contrast agents, such as organic dyes [60-62], genetically encoded proteins [63-66], and micro/nanoparticles [67-71], have been extensively explored for PA molecular imaging. With the innovations and advances in exogenous contrast agents, molecular PAT has recently enjoyed a prosperous development (Figure 4).

A tyrosinase-based reporter that triggers human cells to express eumelanin has been developed to provide a strong PA contrast [64]. Combining the tyrosinase-based reporter and a Fabry-Perot-based ultrasound sensor (22 MHz bandwidth), PACT has imaged transgenic cancer cells expressing eumelanin (Figure 4(a), illumination wavelength: 600 nm) and monitored tumor development *in vivo* [64]. An *in vivo* pH mapping technology, consisting of a photoacoustic pH indicator, SNARF-5F, has been developed [72]. Facilitated by multiwavelength PACT, the pH levels inside a tumor have been quantitatively measured. Here, a linear transducer array-based PACT system (128 elements, 11.25 MHz central frequency, and 75% one-way bandwidth) was used. The SNARF-5F-based nanoparticles were injected via the tail vein into a tumor-bearing mouse; multiwavelength PACT of the mouse at 75 min postinjection has mapped the pH distribution inside the tumor *in vivo* (Figure 4(b), illumination wavelengths: 565 nm and 600 nm) [72]. Bacterial outer membrane vesicles (OMVs) have been explored for delivering drugs, vaccines, and immunotherapy agents. Recently, bioengineered OMVs for contrast-enhanced PA imaging have been investigated [73]. OMVs encapsulating biopolymer-melanin (OMV^Mel^) were produced using a tyrosinase-expressing bacterial strain, which provides strong optical absorption at NIR wavelengths. Here, an arc-shaped transducer array-based PACT (270° in-plane detection angle, 256 elements, 5 MHz central frequency, and 90% one-way bandwidth) was used. Using multiwavelength PACT, tumor-associated OMV^Mel^ distribution has been monitored *in vivo* (Figure 4(c), illumination wavelengths: 680-900 nm and 10 nm interval) [73].

Genetically encoded photoswitchable proteins (RpBphP1, DrBphP-PCM, etc.) that effectively improve the PA imaging sensitivity and specificity have been reported recently [31, 32, 63, 65, 66, 74, 75]. Here, a 512-element full-ring transducer array-based PACT system (5 MHz central frequency, 90% one-way bandwidth) was used. By analyzing the unique decay characteristics of different photoswitchable proteins, quantitative multicontrast imaging has been achieved to differentiate multiple types of tumors in deep tissue *in vivo* (Figure 4(d), illumination wavelength: 780 nm) [65, 74]. In addition, a split version of DrBphP-PCM has been engineered, providing the first bimolecular PA complementation reporter to detect protein-protein interactions in deep tissue *in vivo* (Figure 4(e)) [74].

Substantial progress in the development of micro/nanorobots has been accomplished for biomedical applications in recent years. However, present micro/nanorobot platforms are ineffective for imaging and motion control in deep tissue *in vivo*. Recently, a PACT-navigated microrobotic system that achieved controlled propulsion and prolonged cargo retention *in vivo* was reported, where a full-ring transducer array (512 elements, 5 MHz central frequency, 90% one-way bandwidth) was used for acoustic detection [76, 77]. Thanks to the molecular contrast and high spatiotemporal resolution at depths, PACT can locate and navigate the microrobots *in vivo*. As shown in Figure 3(f) (illumination wavelength: 750 nm), PACT visualizes the migration of microrobots in the intestines in real time *in vivo* [76]. The integration of PACT and the newly engineered microrobotic system allows deep tissue imaging and accurate control of the microrobots *in vivo* and paves a new path for precision medicine.

## 4. PAT of the Brain

**Figure 5:**
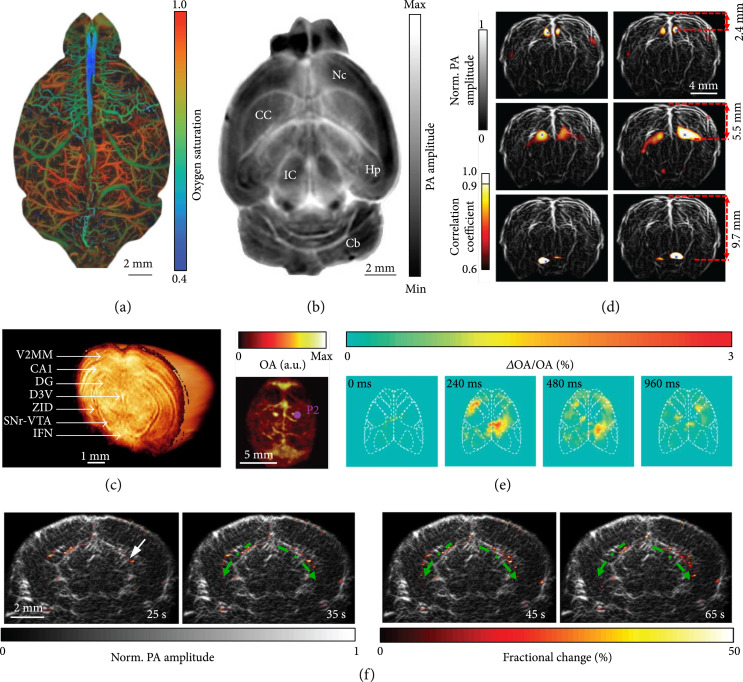
Multiscale PAT of the brain. (a) PAM of oxygen saturation of hemoglobin in a mouse brain [79]. (b) A cross-sectional PACT image of a saline-perfused mouse brain (horizontal plane) at 2.8 mm depth, showing internal structures of the brain clearly. Nc: neocortex; CC: corpus callosum; Hp: hippocampus; Cb: cerebellum; IC: inferior colliculus [80]. (c) 3D PACT image of a mouse brain *ex vivo*. Illumination wavelength: 740 nm; V2MM: secondary visual cortex, medio-medial; CA1: hippocampal CA1 area; DG: dentate gyrus; D3V: dorsal third ventricle; ZID: zona incerta dorsal; SNr: substantia nigra reticulate; VTA: ventral tegmental area; IFN: interfascicular nucleus [81]. (d) Functional mapping of the resting-state connectivity in a rat whole brain (coronal plane), showing a clear correlation between corresponding regions across the left and right hemispheres [58]. (e) PACT of GCaMP6s responses to electrical stimulation of the right or left hind paw. First from the left, maximum amplitude projection along the depth direction of the 3D images of a GCaMP6-expressing mouse; second to last, relative increases in PA signal with respect to the baseline for a slice at ~1 mm depth at different time points following the stimulation pulse for the GCaMP6s-expressing mouse [82]. (f) PACT images of epileptic activities during a seizure at different times. The fractional changes (color) in the PA amplitude are overlaid on the anatomical image (gray, Bregma: −1.0 mm). The arrow indicates the injection site, and the dashed green arrows indicate the epileptic wave propagation direction [83].

Studying how the brain works will directly benefit basic science and help us to better understand and treat neurological disorders, such as Alzheimer’s and Parkinson’s diseases [78]. Thanks to optical contrast and deep penetration, PAT provides a powerful tool for multiscale functional brain imaging. Based on the endogenous molecules, including hemoglobin, lipid, and cytochrome, label-free PAT has resolved whole-brain vasculature and structures. Taking advantage of the high resolution and high sensitivity, PAM has mapped cortical vasculature and quantified oxygen saturation of hemoglobin (sO_2_) at the single capillary level with the skull intact (Figure 5(a), illumination wavelength: 532 nm) [79]. After mitigating the blood signals from the brain, label-free PACT has imaged detailed internal brain structures with MRI image quality. Based on the lipid and cytochrome contrast, a full-ring transducer array-based PACT (512 elements, 5 MHz central frequency, and 90% one-way bandwidth) identified brain structures, including neocortex, corpus callosum, hippocampus, inferior colliculus, and cerebellum (Figure 5(b), illumination wavelength: 600 nm) [80]. Employing NIR light illumination, a hemispherical transducer array-based PACT (512 elements, 140° angular tomographic coverage, 5 MHz central frequency, and 100% one-way bandwidth) has imaged a mouse brain with structural details revealed in 3D *ex vivo* (Figure 5(c), illumination wavelength: 740 nm) [81]. A full-ring transducer array-based PACT (512 elements, 5 MHz central frequency, and 90% one-way bandwidth) has also monitored the whole rat brain resting-state hemodynamics and mapped the whole-brain functional connectivity (Figure 5(d), illumination wavelength: 1064 nm) [58].

The deep penetration and high spatiotemporal resolution enable PACT to monitor large-scale neural activities in detail. As a demonstration, a hemispherical transducer array-based PACT (512 elements, 5 MHz central frequency, and 100% one-way bandwidth) has imaged rapid calcium responses to electrical stimulations in a GCaMP6s expressing mouse brain *in vivo* (Figure 5(e), illumination wavelength: 488 nm) [82]. In addition, a linear transducer array-based PACT (256 elements, 21 MHz central frequency, and 52% one-way bandwidth) has also visualized the epileptic wave propagation across the whole brain during a seizure (Figure 5(f), illumination wavelength: 1064 nm) [83]. With the capability to monitor neuronal activities and hemodynamics across the whole brain, PAT has displayed encouraging potentials in studying various brain disorders and diseases, such as traumatic disorders, brain cancer, stroke, Alzheimer’s disease, and seizures of various etiologies.

## 5. Broad Spectrum PAM

Any molecule has a unique absorption spectrum and has a fluorescence quantum yield lower than 100%, providing contrast for PAT [27]. By tuning the wavelength from ultraviolet (UV) to mid-infrared (MIR), spectral PAT has detected endogenous biological molecules, including cytochromes, DNA/RNA, hemoglobin, myoglobin, melanin, protein, lipids, and water [12, 19, 20, 84-88].

**Figure 6:**
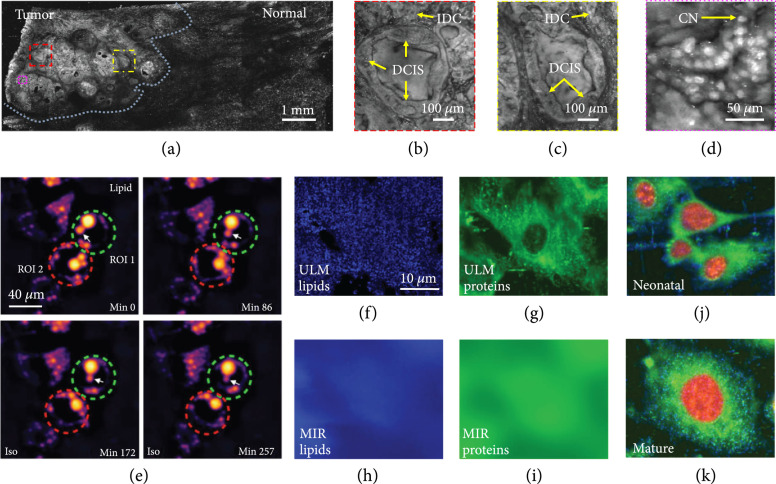
Broad-spectrum PAM of tissues and cells. (a) PAM image of a fixed, unprocessed breast tumor. Illumination wavelength, 266 nm [88]. (b-d) Zoomed-in PAM images of the red, yellow, and magenta dashed regions in (a), respectively. IDC: invasive ductal carcinoma; DCIS: ductal carcinoma in situ; CN: cell nuclei [88]. (e) Monitoring intrinsic lipid contrast during lipolysis in differentiated 3T3-L1 adipocytes at 2,857 cm^−1^. Two regions of interest (ROIs) enclosing individual adipocytes are marked: green dashed circle for ROI 1 and red dashed circle for ROI 2. The white arrow follows the process of lipid droplet remodeling in a single adipocyte enclosed in ROI 1 [91]. (f, g) ULM-PAM images of lipids (f) and proteins (g) [85]. MIR-PAM images of lipids (h) and proteins (i), imaged at 3,420 nm and 6,050 nm, respectively [85]. Composite images of cells formed by overlaying the images of lipids (blue), proteins (green), and nucleic acids (red) in different color channels at neonatal (j) and mature (k) stages [85].

Breast-conserving surgery is aimed at excising all cancer cells. However, no intraoperative device that can quickly examine the lumpectomy specimen at a microscopic scale is available. Thus, up to 60% of patients have to undertake second surgeries to reach clear margins. PAM employing UV illumination (UV-PAM) can specifically highlight cell nuclei, yielding microscopic images with a similar contrast as hematoxylin-labeled histological images [20]. A fast UV-PAM system was recently developed to provide high-resolution histology-like images of unprocessed and unlabeled breast tissues [88]. Figure 6(a) shows UV-PAM images of a fixed, unprocessed breast tumor specimen with a field of view (FOV) of 10 mm×4.2 mm (illumination wavelength, 266 nm). The close-up images (Figures 6(b) and 6(c)), corresponding to the red and yellow dash boxed regions in Figure 6(a), reveal detailed structures of the carcinoma. As shown in Figure 6(d) (a zoomed-in image of the magenta dash boxed region in Figure 6(a)), it is clear UV-PAM can resolve individual cell nuclei. Label-free UV-PAM with histology-like imaging capability has demonstrated its potential as an intraoperative margin assessment tool for surgeons and pathologists to identify tumor margins [88].

Conventional optical microscopy is fundamentally limited by a lack of chemical specificity or by cellular phototoxicity; thus, label-free optical imaging cannot directly reveal biomolecule dynamics in living cells [89, 90]. Recently, MIR-PAM, based on chemically specific vibrational excitation by MIR absorption, has been developed to provide label-free bond-selective metabolic imaging in live cells [91]. MIR-PAM enables spatiotemporal profiling of lipids, proteins, and carbohydrates in cells and tissues. As a demonstration, MIR-PAM monitored lipid and protein dynamics during lipolysis in live cells (Figure 6(e), illumination wavelength: 3500 nm), proving its capability of imaging of biomolecular dynamics in living cells without labeling [91].

However, the long MIR wavelength fundamentally limits the spatial resolution of MIR-PAM due to optical diffraction [91]. In addition, the high water content in fresh samples severely reduces the imaging contrast due tot its strong MIR absorption. A recent innovation that employs MIR PA imaging localized with a pulsed UV illumination (266 nm) successfully overcomes the above limitations. This technology, termed ultraviolet-localized MIR PAM (ULM-PAM), has achieved high-resolution MIR imaging of fresh samples without water background [85]. ULM-PAM employed a focused mid-MIR laser pulse to excite the sample. A confocally aligned UV laser pulse photoacoustically detected the MIR-induced temperature rise, thereby revealing MIR absorption contrast. ULM-PAM’s lateral resolution, defined by the UV wavelength, is over 10-fold higher than conventional MIR microscopy. Moreover, most biomolecules in living cells, including lipids, proteins, and nucleic acids, have strong absorption of UV light (200-230 nm). Meanwhile, UV light is transmissive in water, which significantly suppresses the water background in ULM-MIR. Illuminating formalin-fixed 3T3 mouse fibroblast cells at wavelengths of 3420 nm and 6050 nm, respectively, ULM-PAM mapped detailed distribution of lipids and proteins in living cells (Figures 6(f) and 6(g)) [85]. As a comparison, the MIR-PAM images of lipids (Figure 6(h)) and proteins (Figure 6(i)) display a high water background and low spatial resolution [85]. As a demonstration, ULM-PAM has imaged neonatal (Figure 6(j)) and mature (Figure 6(k)) cells, showing high-resolution, high-contrast imaging of lipids (blue), proteins (green), and nucleic acids (red) [85]. Therefore, ULM-PAM permits label-free imaging of biological samples at high resolution with high contrast.

## 6. PACT of Human Breasts

**Figure 7:**
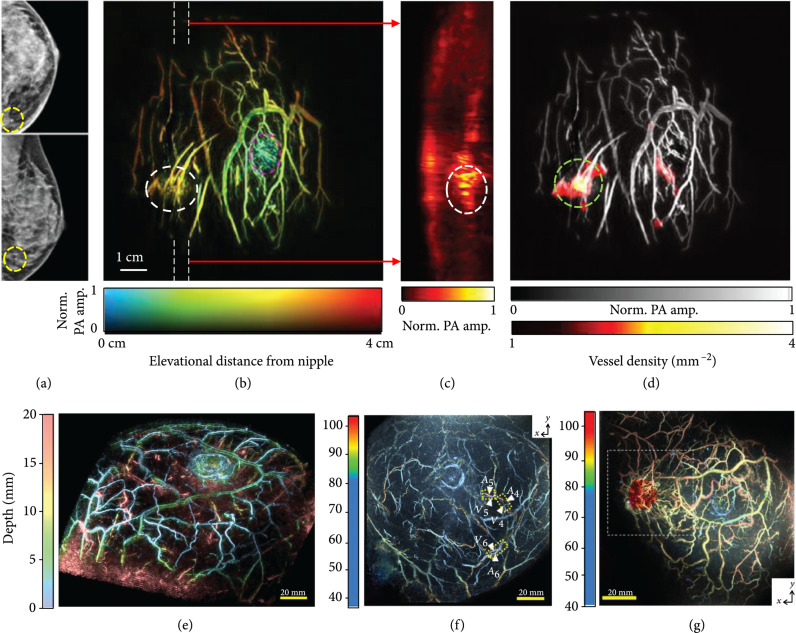
PACT of human breasts. (a) X-ray mammograms of an affected breast [94]. (b) Depth-encoded angiogram of the affected breast acquired by SBH-PACT. The breast tumor is identified by a white circle; the nipple is marked by a magenta circle [94]. (c) Maximum amplitude projection (MAP) images of the thick slice in sagittal planes marked by white dashed lines in (b) [94]. (d) Automatic tumor detection on vessel density maps. Tumors are identified by green circles. Background images in gray scale are the MAP of vessels deeper than the nipple [94]. (e) Depth-encoded 3D PACT image of a healthy breast [95]. (f) Evaluation of the S-factor in a healthy breast, where the color represents the measured S-factor [96]. (g) A fusion image of the S-factor and 3D-US images (red color) [96].

Breast cancer is the number two cause of cancer death globally (11.6%), with a worrying mortality rate of 6.6% [92, 93]. Recently, substantial progress has been made for noninvasive breast cancer diagnosis utilizing PAT. A technical advance, termed single-breath-hold PACT (SBH-PACT), can obtain a 3D image of a whole breast within a single breath hold (~15 s) [94]. SBH-PACT employed a full-ring transducer array (512 elements, 2.25 MHz central frequency, and 95% one-way bandwidth) for acoustic detection and 1064 nm light for excitation. The high detection sensitivity of SBH-PACT allows detecting breast tumors in detail, promising wide applications in clinical breast care. By detecting local angiogenesis, SBH-PACT can differentiate lesions from normal tissues. Highly correlated with the tumor sites indicated in X-ray mammograms (Figure 7(a)), SBH-PACT can identify a tumor by revealing its higher blood vessel densities (Figure 7(b)) [94]. By further examining a tumor-containing slice (marked by white dashed lines in Figure 7(b)), the same tumor, displaying higher PA signals, can be visualized at the corresponding location (Figure 7(c)) [94]. Furthermore, a vessel density map of the breast was computed, where the breast tumor was highlighted due to its high vessel density (Figure 7(d)) [94].

A PACT system with spiral scanning of a hemispherical transducer array (512 elements, 2 MHz central frequency, and 90% one-way bandwidth) was recently reported for human breast imaging [95]. The dense spatial sampling and the isotropic 3D resolutions yielded high-quality imaging of a healthy breast (Figure 7(e), illumination wavelength: 795 nm). By illuminating the breast tissue at two wavelengths of 756 nm and 797 nm, respectively, hemoglobin oxygen saturation (sO_2_) was also evaluated using an approximate parameter, termed S-factor, computed from measurements obtained at the two wavelengths [96]. The S-factors can be evaluated on neighboring vessels, such as adjacent arteries and veins, assuming that the optical fluence in the neighboring region is the same. As shown in Figure 7(f), arteries and veins in a healthy breast can be clearly distinguished in the S-factor image [96]. Figure 7(g), a fusion of the ultrasound (red color) and S-factor images, shows an example of the results obtained from a breast cancer lesion. Notably, the detailed vasculature surrounding the tumor is clearly visible. In addition, the arterioles and venules show clustering [96].

Recent advances in PACT for breast imaging hold the potential to complement X-ray mammography for breast cancer diagnosis and treatment monitoring. Unlike X-ray mammography, PACT uses light for excitation, which is nonionizing and safe. In the meantime, it provides sufficient penetration in the breast. Moreover, the optical absorption provides a much higher soft tissue contrast than the X-ray mammography. Further, PACT can noninvasively monitor breast tumor responses to chemotherapy as treatment proceeds.

## 7. PACT of Human Extremities

Vascular disease is the leading cause of death in the United States (~30%) [96]. And vascular disease most commonly presents in appendicular regions. In addition, peripheral blood vessel examination can identify the visceral disease and estimate an individual’s lifestyle [97]. Therefore, angiographic imaging of extremities can offer significant insights into the health conditions of patients, especially for hypertensive, diabetic, and hyperlipidemic patients [96]. Thanks to its high sensitivity of blood, deep tissue penetration, and noninvasiveness, PACT becomes a promising modality for imaging the vasculature of extremities.

**Figure 8:**
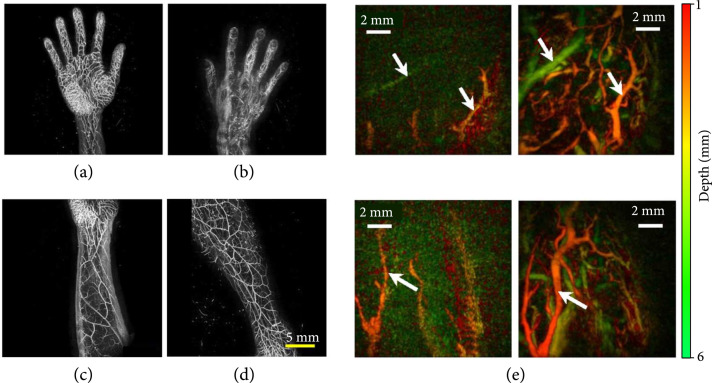
PACT images of human extremities. (a-d) PACT images of various extremities, including palm (a), back of the hand (b), forearm (c), and lower thigh [98]. (e) MAP images of human fingertips after cold (left-hand panels) and warm (right-hand panels) water immersion, color-coded for depth, while arrows show the same vessels in each imaging condition [99].

A newly designed PACT system equipped with a hemispherical transducer array (1024 elements, 3.34 MHz central frequency, and 85% one-way bandwidth) was developed to image the vasculature of human limbs [98]. After scanning the detector array, it can image a field of view up to 180 mm×270 mm within 10 minutes, providing high-quality images of human limbs. As shown in Figures 8(a)-8(d) (illumination wavelength, 797 nm), various extremities, including palm (Figure 8(a)), back of the hand (Figure 8(b)), forearm (Figure 8(c)), and lower thigh (Figure 8(d)), have been imaged, revealing detailed vasculature [98].

Recently, a PACT system with a Fabry-Perot interferometric ultrasound sensor (30 MHz bandwidth, -3 dB points) was built [99]. A volumetric image (14×14×14 mm^3^) can be acquired within 90 s at a 30 Hz laser repetition rate (illumination wavelength, 750 nm). Fingertips were imaged before and after a thermal stimulus. As shown in Figure 8(e), fewer vessels were depicted after cold water immersion, showing thermally induced peripheral vasoconstriction [99].

These experiments demonstrated the capability of PACT to image peripheral vessels and their responses to vasomotor changes, promising the diagnosis of peripheral vascular diseases.

## 8. Photoacoustic Topography through an Ergodic Relay

In previous sections, we review the advanced technical innovations and their widespread applications of both PAM and PACT. However, till now, PAM still requires serial detection using a focused detector for data acquisition, which limits the throughput, while PACT employs parallel detection using multiple detection elements and multichannel amplifiers and digitizers, which is complex and expensive. Recently, a high-throughput PA imaging technique based on an ergodic relay (ER) has been developed, which employs a single-element detector to obtain snapshot wide-field images. This technology is termed as photoacoustic topography through an ER (PATER) [100]. The ER, a critical component in PATER, is a waveguide that permits acoustic waves originated from any input point to reach any other output point with distinctive reverberant characteristics [101]. Here, the ER effectively encodes each one-dimensional depth image into a unique temporal sequence. Because of the uniqueness of each temporal signal, PA waves from the whole volume through the ER can be recorded in parallel. Finally, we can decode them mathematically to reconstruct 2D projection images.

**Figure 9:**
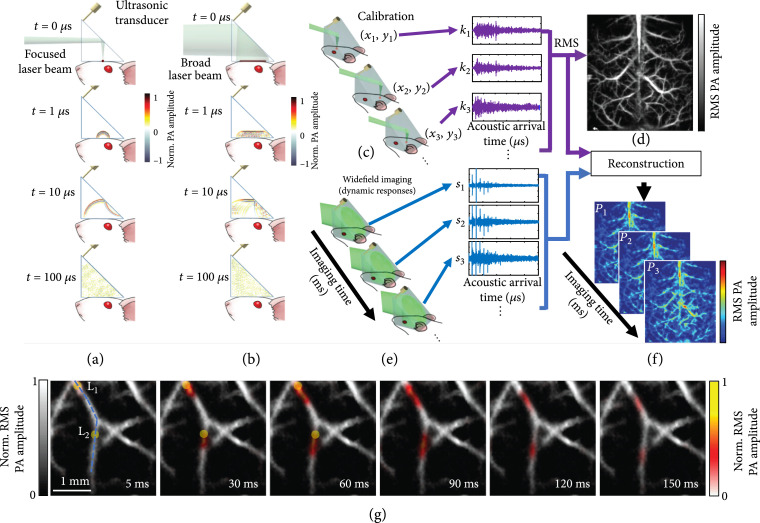
Principles of PATER and representative images [100]. (a-f) Principles of PATER. (a) Simulation of acoustic propagation in the ER in calibration mode. Norm.: normalized. (b) Simulation of acoustic propagation in the ER in wide-field mode. (c) In the calibration mode, light is focused on each pixel to acquire the impulse response encoded by the ER, $k_{i}$, and then raster scanned over the FOV. (d) Calibration image formed by computing the root-mean-squared amplitude of each received PA signal at every calibration position. (e) Snapshot wide-field imaging. A broad laser beam illuminates the entire FOV to acquire encoded signals, $s_{i}$, which can be repeated for high-speed imaging. (f) Reconstructed wide-field images. The reconstruction algorithm uses calibrated impulse responses to decode the wide-field signals and then display wide-field images. (g) Monitoring of blood pulse wave propagation. Wide-field images at different time points illustrate the thermal wave propagation in the middle cerebral arteries. The yellow circles, labeled $L_{1}$ and $L_{2}$, indicate the locations of the laser heating spots during wide-field measurement. The thermal wave signals are shown in color, and the background vessels are shown in gray.

The mechanism of PATER has been illustrated in Figures 9(a)-9(f). PATER has two imaging modes: the calibration mode (Figure 9(a)) and the wide-field mode (Figure 9(b)). In the calibration mode, a focused laser pulse illuminates the object. The PA wave excited by the focused laser pulse can be treated as a spatiotemporal delta function of the PATER system. An unfocused needle transducer (20 MHz central frequency, 56% one-way bandwidth) was used for acoustic detection. Each calibration acquisition measures the impulse response of the system at each pixel. After raster scanning the focus across the entire FOV, the whole system’s impulse responses are obtained (Figure 9(c)). By computing the root-mean-squared amplitude of the PA signal received at each pixel, PATER forms a projection image of the object (Figure 9(d)). In the wide-field mode, a broad laser beam excites the whole FOV. PA waves from the whole volume are detected in parallel, allowing high-speed snapshot wide-field imaging (Figure 9(e)). By solving an inverse problem, PATER reconstructs wide-field images (Figure 9(f)).

Because of the high propagation speed of blood pulse waves in major arteries, visualization of pulse wave propagation is still challenging. Thanks to PATER’s high imaging speed, real-time imaging of pulse wave propagation *in vivo* has been achieved. After calibration, wide-field measurements the mouse brain were taken. A laser diode generated two focal spots to heat the middle cerebral arteries (MCAs) during the wide-field recording. The wide-field images (Figure 9(g), illumination wavelength: 532 nm) reveal thermal wave propagation with the blood flow in the MCAs. By analyzing the time-lapse images, we can compute the pulse wave velocity (PWV) in major arteries, a key physiological parameter indicating cardiovascular disease progression. PATER facilitates imaging of fast dynamics *in vivo* and holds great potentials for wide ranging biomedical applications—such as high-speed imaging of neural action potentials and high-throughput analysis of histological tissues.

## 9. Machine Learning-Based Image Reconstruction

Machine learning and deep learning approaches have prompted a paradigm shift in biomedical imaging [102, 103]. In PAT, various image reconstruction algorithms based on physics have been studied [104-108]; the recent development of machine learning (ML) offers new possibilities for advanced reconstruction and processing methods. For example, ML methods have been used to identify cancer lesions by feature learning of texture patches [109]. Convolutional neural networks (CNNs) have been explored to remove streak-type artifacts from the PACT images [110-112] and applied to quantitative spectral unmixing of multiple chromophores in deep tissue [113]. Here, we mainly focus on two major applications—reconstruction with sparse data and limited-view PACT.

**Figure 10:**
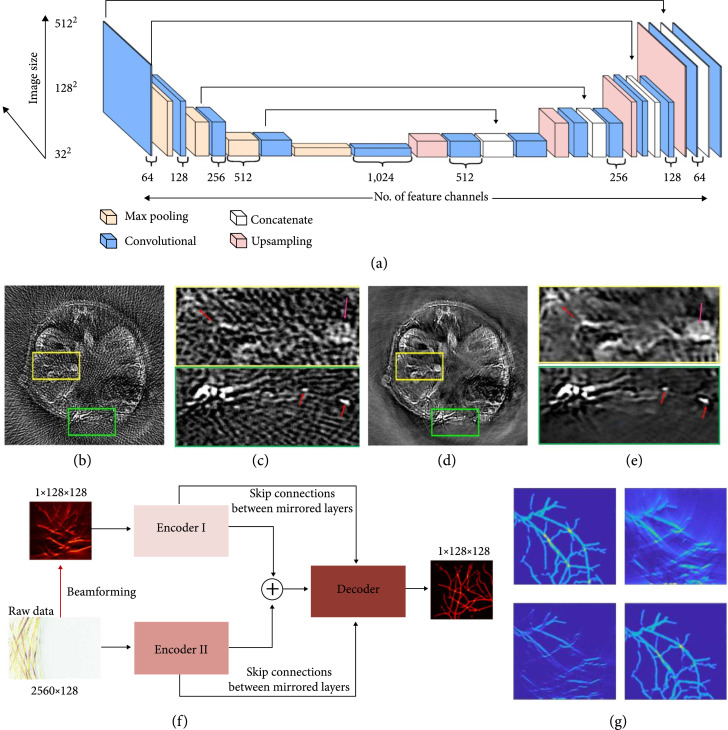
Image reconstructions assisted by machine learning. (a-e) Deep learning PAT with sparse data [114]. (a) The U-Net network architecture, consisting of contracting (downsampling) and expansive (upsampling) paths, which is used for the image reconstruction with sparse data. (b) Artifactual reconstructed image with undersampled (128 projections) data, showing the reconstruction artifacts due to the sparse data. (c) Zoom-in images of the yellow and green boxed regions in (b). (d) Artifact-free counterpart of (b), obtained with the trained network. (e) Zoom-in images of the yellow and green boxed regions in (d). (f, g) Hybrid neural network for limited-view PACT. [117] (f) The global architecture of Y-Net. Two encoders extract different input features, which concatenate into the decoder. Both encoders have skip connections with the decoder. (g) Comparison of reconstructed images. Top left, ground truth; top right, image reconstructed using the universal back-projection method; bottom left, image reconstructed using the time-reversal method; bottom right, image reconstructed using the trained Y-net.

In PACT, strategies for fast data acquisition usually involve suboptimal spatial or temporal sampling of the raw channel data, leading to a trade-off between the imaging quality and speed. Recently, a deep CNN-based framework for high-performance image reconstruction based on sparsely-sampled PA data was proposed [114]. The U-Net architecture (Figure 10(a)) was trained to recognize the artifacts from sparse data reconstruction [114]. Then, the trained network was applied to *in vivo* imaging. Mice were imaged by a PACT scanner equipped with a full-ring transducer array consisting of 512 elements (5 MHz central frequency, 80% one-way bandwidth). The reconstructed images with all 512-channel data were used as the ground truth for training. Results of the artifact removal from cross-sectional images reconstructed with 128-channel data are shown in Figures 10(b)-10(e) (illumination wavelength, 1064 nm), respectively [114]. The internal structures of the mouse, which can barely be resolved in the original reconstruction (Figure 10(b)) due to the overwhelming artifacts, have been revealed after the network correction (Figure 10(d)) [114]. The reconstruction algorithm can differentiate the small blood vessels from the streak artifacts (labeled by red arrows in the close-up panels in Figures 10(c) and 10(e)) and selectively suppress the artifacts [114].

Linear transducer array-based PACT often suffers from limited-view imaging [115, 116]. Recently, a hybrid neural network was proposed to address this issue. The hybrid neural network, Y-Net, is a CNN framework to reconstruct PA images. Y-Net combines the raw channel data and beamformed images as input [117]. As illustrated in Figure 10(f), the Y-Net connects two encoder paths with one decoder path, utilizing the information from channel data and beamformed images. Encoder I encodes the texture features, while Encoder II encodes the physical features. The decoder concatenates the two encoder outputs and yields the final images. Y-Net’s performance was tested by numerical simulations (Figure 10(g)). Comparing with the ground truth image (Figure 10(g), top left panel), the images reconstructed using conventional methods, including universal back-projection (Figure 10(g), top right panel) and time reversal reconstruction (Figure 10(g), bottom left panel), reveal missing vertical features due to the limited view issue. The Y-Net output successfully mitigates the problem and reconstructs an image much closer to the ground truth [117].

## 10. Outlook

The rapidly growing applications of PAT in basic life science study and clinical translation provide strong momentum for the continuous development of PAT. The recent advances in small-animal imaging promise whole-brain monitoring of neural activities. For visualizing neural responses in the deep brain, new NIR calcium- or voltage-sensitive indicators with performance optimized for PAT need to be engineered. The tremendous progress in genetically encoded PA probes enables noninvasive tracking of tumor growth and metastasizing, providing a powerful tool to better understand the tumor and find an effective treatment. The elegant marriage between PAT and the microrobotic system permits real-time navigation of the microrobots and promises precision medicine.

The high-impact applications of human breast and extremity imaging boost a fast expansion of PAT in clinical translations. PAT provides a noninvasive and powerful approach for breast cancer diagnosis, chemotherapy monitoring, and peripheral vascular imaging for diabetic patients, complementing current clinical methods in contrast mechanism, spatiotemporal resolution, and penetration. Currently, photon dissipation restricts the ultimate light penetration to ~10 cm in mammalian tissue, which prevents whole-body imaging of human adults using PAT. A potential solution is to employ microwaves for excitation. Biological tissues are more transparent to microwaves, which holds the promise to penetrate beyond 10 cm and permit visualization of internal organs in adults or whole neonatal bodies.

The innovation of PATER offers a solution for low-cost high-throughput PA imaging, enabling the miniaturization of the PAT system toward portable and wearable applications. Particularly, PATER is envisaged to be used as a wearable device to monitor human vital signs. ML-based PA image reconstruction and image processing methods enjoy the latest development and achievements from CNN, providing a novel perspective to address the artifacts in image reconstruction and enhance the performance of PAT. With the recent advances in PAT, we expect more widespread and impactful applications in fundamental science, preclinical research, and clinical translation.
